# Development of an international scale of socio-economic position based on household assets

**DOI:** 10.1186/s12982-015-0035-6

**Published:** 2015-09-22

**Authors:** John Townend, Cosetta Minelli, Imed Harrabi, Daniel O. Obaseki, Karima El-Rhazi, Jaymini Patel, Peter Burney

**Affiliations:** National Heart and Lung Institute, Imperial College, Emmanuel Kaye Building, 1b Manresa Road, London, SW3 6LR UK; Faculté de Médecine, Sousse, Sousse, Tunisia; Department of Medicine, Obafemi Awolowo University, Ile-Ife, Nigeria; Laboratoire d’épidémiologie, Recherche Clinique et Santé Communautaire, Faculté de Médecine de Fès, Université SidiMohammed Ben Abdellah, Fez, Morocco

**Keywords:** Poverty, Measurement tool development, Socio-economic position, Developing countries, Respiratory diseases

## Abstract

**Background:**

The importance of studying associations between socio-economic position and health has often been highlighted. Previous studies have linked the prevalence and severity of lung disease with national wealth and with socio-economic position within some countries but there has been no systematic evaluation of the association between lung function and poverty at the individual level on a global scale. The BOLD study has collected data on lung function for individuals in a wide range of countries, however a barrier to relating this to personal socio-economic position is the need for a suitable measure to compare individuals within and between countries. In this paper we test a method for assessing socio-economic position based on the scalability of a set of durable assets (Mokken scaling), and compare its usefulness across countries of varying gross national income per capita.

**Results:**

Ten out of 15 candidate asset questions included in the questionnaire were found to form a Mokken type scale closely associated with GNI per capita (Spearman’s rank r_s_ = 0.91, p = 0.002). The same set of assets conformed to a scale in 7 out of the 8 countries, the remaining country being Saudi Arabia where most respondents owned most of the assets. There was good consistency in the rank ordering of ownership of the assets in the different countries (Cronbach’s alpha = 0.96). Scores on the Mokken scale were highly correlated with scores developed using principal component analysis (r_s_ = 0.977).

**Conclusions:**

Mokken scaling is a potentially valuable tool for uncovering links between disease and socio-economic position within and between countries. It provides an alternative to currently used methods such as principal component analysis for combining personal asset data to give an indication of individuals’ relative wealth. Relative strengths of the Mokken scale method were considered to be ease of interpretation, adaptability for comparison with other datasets, and reliability of imputation for even quite large proportions of missing values.

## Background

Studying associations between health outcomes and socio-economic position is an important aspect of health research [1]. A measure of socio-economic position may be useful to describe and monitor the social distribution of disease to inform health policy, to explain causal mechanisms through which socio-economic position generates health differences, or to statistically adjust for socio-economic circumstances when another exposure is the main focus of interest [2].

The Burden of Obstructive Lung Disease (BOLD) study is an international survey of ventilatory function [3] which has demonstrated a strong ecological association between “low” forced vital capacity, a measurement of the functional lung size, and gross national income (GNI) per capita, especially in poorer countries [4]. In the United Kingdom associations have been recorded between mortality rates from Chronic Obstructive Pulmonary Disease (COPD) and measures of social class, [5] and of childhood deprivation [6]. Other studies have also linked ventilatory function to different measures of socio-economic status within countries [7]. However, there has so far been no systematic evaluation of lung function and socio-economic position at the individual level on a global scale.

A barrier to achieving this is the lack of an easily applicable measure of socio-economic position that is broadly relevant to many countries and that discriminates between individuals as well as between regions or countries. No method will capture all aspects of socio-economic position but asset-based measures have been widely used in low and middle income countries because they provide an easily collected variable that is stable over short term economic fluctuations [8]. The usual method of analysing these measures is to use principal component analysis (PCA) to identify complex scores from correlated variables. An alternative is to use simpler approaches such as Mokken scale analysis, which is based on a count of assets selected and ranked for their ability to discriminate between different levels of affluence [9].

In order to establish a measurement of socio-economic position that could be used to compare individuals within and between countries, we used a Mokken scale approach to develop scores using an inventory of assets included in the BOLD study. This was comprised of commonly used items selected from a number of survey instruments and from experience in an attempt to cover a wide range of levels of affluence with relatively few items. In this analysis we examined whether the answers were scalable, i.e. whether the assets tend to be acquired in the same order by different people and all people with a given number of assets tend to have the same set of assets [10]. We compared the Mokken scale with PCA, and checked the scalability in different sites and for both current asset ownership and reported household asset ownership in early life (around 5 years of age). We also tested the face validity of the scale by comparing the mean values obtained in each centre against the GNI per capita associated with each centre and by comparison with other individual and household level variables expected to be associated with socio-economic position.

## Methods

### Overview of the data

The BOLD study has been described in detail elsewhere [3]. Briefly, this is a cross-sectional study of representative samples of adults aged over 40 years old living in centres selected in all the regions of the world defined by the Global Burden of Disease programme, with the exception of the Latin America and the Asian Pacific High Income regions. The current analysis includes only the eight centres, from eight different countries, entering the study after 2006 when new questionnaires were introduced that included 15-item asset inventories relating to the current time and to the time when the participant was 5 years old. Fourteen of the asset questions had binary (yes/no) responses. The respondents were asked whether their household has any of the following: electricity, flush toilet, fixed phone, cell phone, television, radio, refrigerator, car, moped/scooter/motorcycle, washing machine, owns their own home, indoor bath or shower, indoor tap, or an outdoor tap of their own. One further question, how often anyone in their household goes hungry due to lack of money, was recoded from a six point scale to a binary response (never or sometimes). Don’t know responses were considered as missing. Items included in this study were selected to cover a range of values from basic assets such as electricity and radios to luxury goods such as washing machines and cars, all of which were considered to be desirable to most people.

A total of 8910 subjects were randomly selected in the eight countries. Of these 1863 did not provide responses, mainly due to unavailability or untraceability (51 %). Other reasons were refusal to participate (22 %), deceased (0.5 %), ineligible due to age (7 %) or incomplete data collection (19 %). Sixty-one (0.9 %) out of the 7047 respondents had a missing response to at least one of the asset questions and were also excluded from the analyses. Characteristics of those included are given in Table 1. Four hundred and five (6 %) of the respondents had incomplete data for childhood ownership of the selected assets (excluding cell phone) and were also excluded from the comparison with asset ownership scores at the age of five.

**Table 1:** Characteristics of the samples and GNI per capita for the countries included in the study

	Annaba (Algeria)	Fes (Morocco)	Ife (Nigeria)	Penang (Malaysia)	Riyadh (Saudi Arabia)	Sousse (Tunisia)	Srinagar (India)	Tirana (Albania)	Total
Included in random selection (number)	969	985	1704	1217	936	799	1100	1200	8910
Included in this study (number)	886	955	1083	693	758	716	927	968	6986
Sex									
Male	442 (49.9 %)	412 (43.1 %)	406 (37.5 %)	355 (51.2 %)	411 (54.2 %)	331 (46.2 %)	499 (53.8 %)	486 (50.2 %)	3342 (47.8 %)
Female	444 (50.1 %)	543 (56.9 %)	677 (62.5 %)	338 (48.8 %)	347 (45.8 %)	385 (53.8 %)	428 (46.2 %)	482 (49.8 %)	3644 (52.2 %)
Age									
40–54 years	538 (60.7 %)	474 (49.6 %)	522 (48.2 %)	347 (50.1 %)	544 (71.8 %)	416 (58.1 %)	603 (65.0 %)	503 (52.0 %)	3947 (56.5 %)
55–69 years	295 (33.3 %)	363 (38.0 %)	369 (34.1 %)	305 (44.0 %)	206 (27.2 %)	254 (35.5 %)	248 (26.8 %)	351 (36.3 %)	2391 (34.2 %)
≥70 years	53 (6.0 %)	118 (12.4 %)	192 (17.7 %)	41 (5.9 %)	8 (1.1 %)	46 (6.4 %)	76 (8.2 %)	114 (11.8 %)	648 (9.3 %)
GNI per capita ($US)	12,860	6160	4930	22,530	53,760	9680	4750	9950	

### Mokken scaling

We first developed a scale using item response theory based on the idea that the items formed a hierarchy such that ownership of any of the assets implied ability to own all the items lower in the order. The method used was a non-parametric model attributed to Mokken [11] which differs from the earlier Guttman-type scale [12] in that it is probabilistic in nature, and from similar but parametric models (e.g. the Rasch scale [13]) in that it makes no assumptions about the shape of the relationship between the probability of item ownership and the trait being measured (such as socio-economic position).

Mokken scales are defined by three fundamental assumptions [14]: (1) unidimensionality (responses to all of the items are explained by a common trait); (2) local independence (ownership depends on the trait but not on ownership of other items in the scale); (3) monotonic increase (or decrease) in the probability of owning each of the assets with increases in the trait being measured. Scalability of a set of items is assessed using Loevinger’s H coefficients, which relate the number of times high-order assets are owned without ownership of all of the lower assets, to the frequency with which this would occur by chance. Loevinger’s coefficients can be calculated for the fit of each individual item in the scale (H_j_) and for the scale as a whole (H). The usual interpretation is that 0.3 ≤ H<0.4 implies a weak scale, 0.4 ≤ H<0.5 a medium scale and ≥0.5 a strong scale, whilst scales with H < 0.3 do not satisfy a Mokken scale [14, 15].

### Selection of items for the Mokken scale

Data from all of the countries combined and an automated item selection procedure (AISP) were used to select suitable items for inclusion in the scale. This procedure successively adds items to the scale such that the whole scale H coefficient and the individual item, H_j_ coefficients were ≥0.3. The procedure stops when no more items meeting these criteria could be added [14].

### Applicability of the scale in different countries

To examine the scalabilty of the selected assets in different countries, item (H_i_) and whole scale (H) coefficients were also calculated for each of the individual countries, using the same set of assets in each case. To assess the consistency of the scale across countries, the selected assets were ranked according to the proportion of respondents who owned them in each country. The consistency of the item ownership rankings between the eight countries was assessed using Cronbach’s alpha. Item rankings within countries were also plotted against the equivalent ranks for all of the countries combined.

For each of the selected items an item response curve (IRC) was produced by plotting the probability of an individual owning the asset against the total number of these assets owned. If the total scores are related to an individual’s wealth this could be thought of as a plot of the probability of an individual owning an item against his/her overall wealth. In order to test for differences between the IRCs in different countries [known as differential item functioning (DIF)] each country’s IRC was compared with that for all other countries combined using the Mantel–Haenszel test [16], and the resulting eight p values were adjusted using a Bonferroni procedure. It was considered that there was significant DIF for that asset (i.e. inconsistency between countries) where the minimum of the adjusted p values was less than 0.05. This procedure was repeated for each of the selected items in the scale in turn.

### Comparison with scores for childhood assets

Scores and Loevinger’s H coefficients were also calculated for the same selected set of assets when the respondent was 5 years old, excluding cell phones as these were not available at that time. Only individuals with complete data for childhood ownership of the remaining selected assets were included. Equivalent scores, excluding cell phone, were calculated for the current asset data for comparison. Scores for current and childhood ownership of the assets were compared graphically.

### Comparison of scores with national wealth and variables related to socio-economic position

In order to check whether the scores given by our scale were related to wealth we plotted the individual and country mean scores against the GNI per capita [17] of the country where the respondent lived for the year the survey took place. We also assessed the strength of relationship between country mean scores and GNI per capita using Spearman’s rank correlation coefficient (r_s_). Whilst we did not have any direct measure of individual wealth we assessed the face validity of the scale by determining the direction of the rank correlation between the scores and a number of variables collected for each respondent which we expected to be broadly related to socio-economic position, particularly in low and middle income countries. The variables studied were the highest level of education completed by the respondent and his/her father and mother, height, body mass index (BMI), frequency of someone in the household going hungry for lack of money, and number of people per room in their household.

### Sensitivity analysis

To compare socio-economic position using data collected in different surveys it may sometimes be necessary to exclude items from the scale. In some circumstances it may also be desirable to include the same set of items in all countries, even though they are not related to socio-economic position in all of the populations. To examine the robustness of the scale to these scenarios we plotted cumulative distribution curves for the scores when one of the questions (cell phone) was omitted and also when an additional random item [~Bernoulli (0.5)] was included. We also assessed the usefulness of an imputation procedure for missing values. This procedure first orders the items according to overall prevalence in the data. The algorithm then imputes a positive response for a missing value if the individual gave a positive response for the following (more common) item, else a negative response if he/she gave a negative response for the preceding (less common) item. If the value to be imputed cannot be determined in this way it is calculated from the relative numbers of positive and negative responses the individual gave for higher and lower items in the scale [18]. Five, 10 or 25 % of the responses were deleted at random and replaced by imputed values. In each case the scores in the resulting dataset were then compared with those in the original dataset.

### Comparison with PCA

Finally we used PCA to develop scores from the assets, using both the full list of 15 assets and the 10 assets selected for the Mokken scale. We compared the individuals’ scores on the first principal component derived from these two PCA analyses with the individual Mokken scale scores, and country mean scores for all three methods with GNI per capita, using Spearman’s rank correlation coefficients.

All analyses were carried out using Stata v13.1, (StataCorp, College Station, TX, USA). Selection of items for inclusion in the Mokken scale and imputation of missing values were carried out using the user-written add-on commands in Stata, *msp* and *imputemok* [14, 18].

## Results

**Fig. 1:**
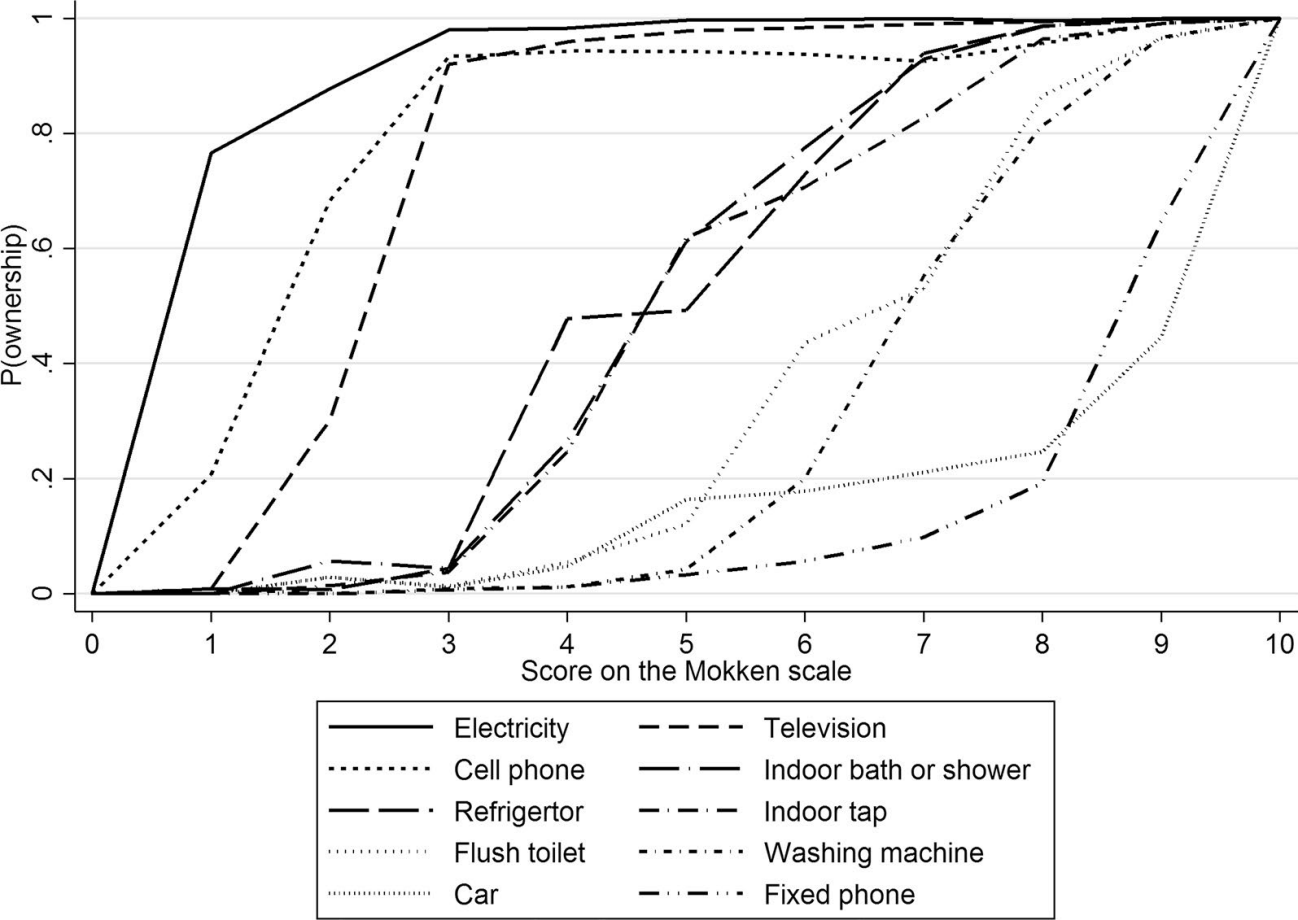
Item response curves for each of the assets included in the Mokken scale

From the original list of 15 assets thought to be related to socio-economic position, the AISP selected 10 assets for inclusion in the Mokken scale (Table 2). Loevinger’s item coefficients (H_j_) were all greater than 0.44 and the overall H coefficient was 0.70, indicating that the items formed a strong scale. This is shown graphically for the selected assets in Fig. 1, which also confirms that the items all had virtually monotonic relationships with the trait being measured.

**Table 2:** Assets selected for the Mokken scale and Loevinger’s H coefficients, ranked by prevalence of ownership

Asset	H_j_ coeff	N	%	Rank
Electricity	0.68	6907	98.9	1
Television	0.73	6678	95.6	2
Cell phone	0.44	6627	94.9	3
Refrigerator	0.70	5619	80.4	4
Indoor bath	0.71	5616	80.4	5
Indoor tap	0.69	5478	78.4	6
Flush toilet	0.72	4682	67.0	7
Washing machine	0.76	4466	63.9	8
Car	0.61	3128	44.8	9
Fixed phone	0.74	3109	44.5	10
Overall (H)	0.70			

The number (N) and percentage of respondents who owned each of the assets are shown. Overall number of respondents = 6986

**Fig. 2:**
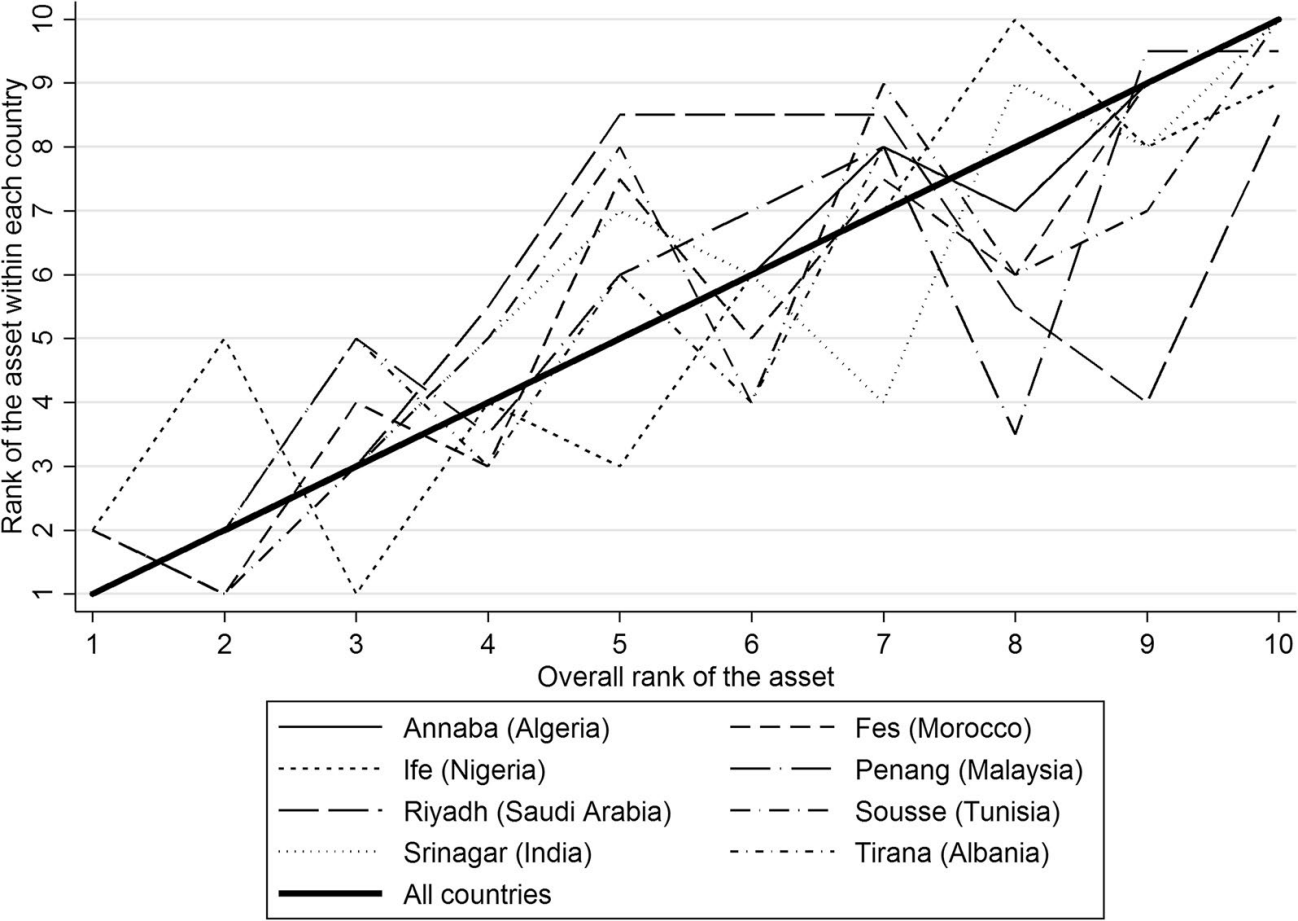
Assets ranked by percentage ownership within each country and overall. Higher ranks signify less commonly owned assets. For overall ownership the items were ranked in the order (*1* most common) electricity, *2* television, *3* cell phone, *4* refrigerator, *5* indoor bath or shower, *6* indoor tap, *7* flush toilet, *8* washing machine, *9* car, (*10* least common) fixed phone

All of the assets showed significant DIF when compared between countries (p < 0.001). Figure 2 shows the rank of each asset in each country plotted against the rank for that asset across all countries. On the whole, both the higher ranked assets (those least commonly owned) and the lowest ranked assets were similar in all countries. This suggests that despite statistically significant differences in individual item functioning, increasing scores on the scale as a whole would generally reflect increasing levels of wealth in all of the countries. Cronbach’s alpha for consistency of the item rankings between countries was 0.96; a very high level of overall consistency. Loevinger’s H_j_ coefficients were only >0.3 for all of the assets in two of the eight countries (Nigeria and Albania), suggesting that some items did not contribute useful information on socio-economic position in some countries. However, overall H coefficients for strength of the scale were >0.3 (i.e. acceptable) for all of the countries except Saudi Arabia.

**Fig. 3:**
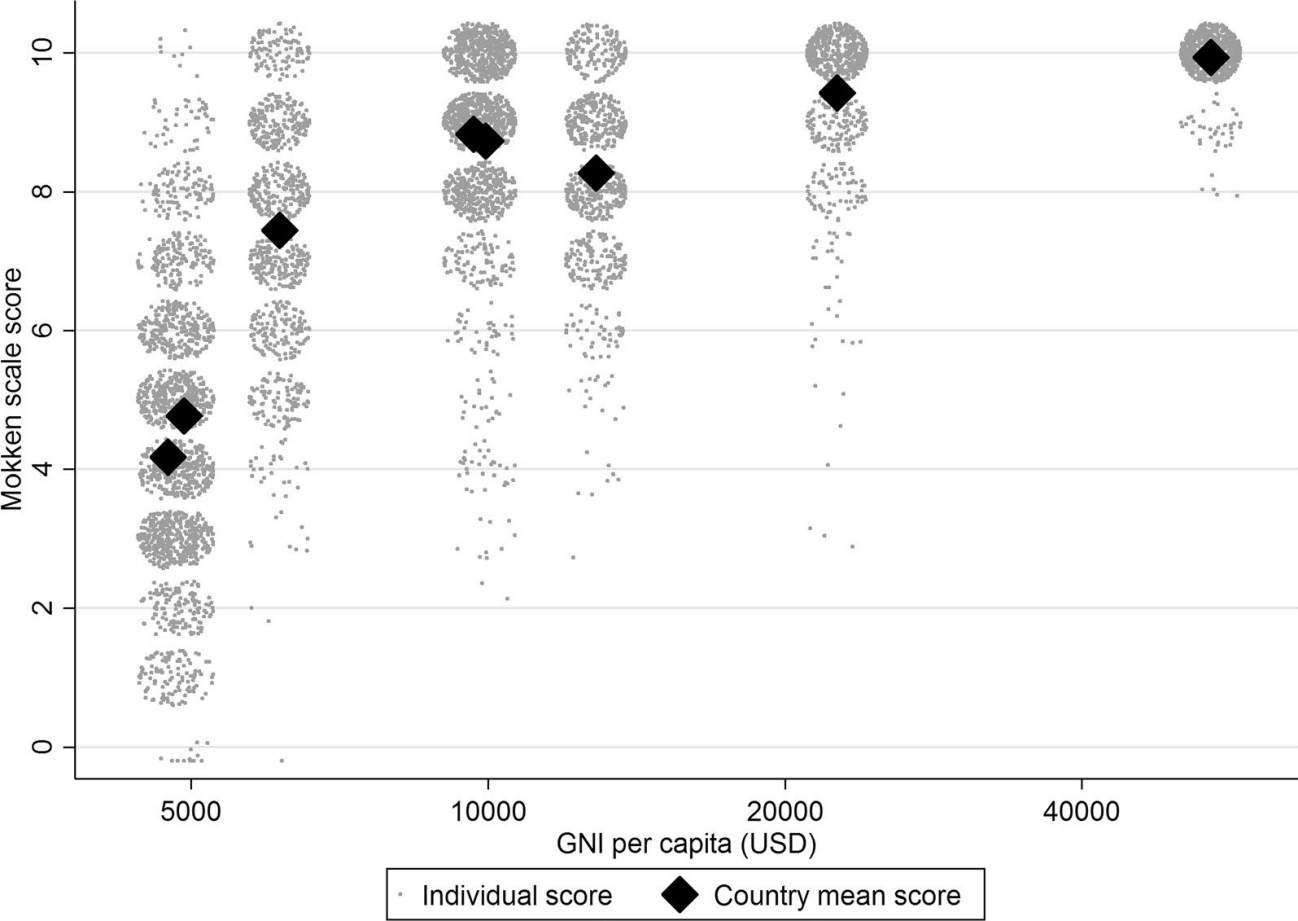
Mokken scale scores vs. GNI per capita for the country the respondent lived in. Some random noise has been added to the individual scores to prevent many points overlying each other

Figure 3 shows the individual and mean scores plotted against the GNI per capita for the country in which the study was situated. There is a clear relation between the mean number of assets owned by individuals and GNI per capita (r_s_ = 0.905, p = 0.002), but with a considerable range of scores in all but the richest countries. When compared with other variables collected for each individual which we expected to be associated with socio-economic position, highest level of schooling was positively and statistically significantly correlated with Mokken scale scores in all countries (p < 0.05) (Table 3). Mother and father’s highest level of education, respondent’s height and BMI were also positively correlated with the scores in most countries and not significantly negatively correlated in any countries. Frequency of someone in the household going hungry for lack of money and number of people per room in the house were negatively correlated with the Mokken scale scores in all of the countries as would be expected for a scale measuring socio-economic position (p < 0.05 in 13 out of these 16 tests).

**Table 3:** Spearman’s rank correlation coefficients (r_s_) for correlations between Mokken scale scores and the stated variables

	Annaba (Algeria)	Fes (Morocco)	Ife (Nigeria)	Penang (Malaysia)	Riyadh (Saudi Arabia)	Sousse (Tunisia)	Srinagar (India)	Tirana (Albania)
Highest level of schooling^a^								
Respondent	0.251*	0.388*	0.411*	0.176*	0.114*	0.301*	0.246*	0.524*
Father	0.161*	0.149*	0.217*	0.147*	0.056	0.149*	0.220*	0.441*
Mother	0.068*	0.066*	0.182*	0.184*	–0.014	0.190*	–	0.405*
Height	0.023	0.038	0.139*	0.123*	0.073	0.050	0.212*	0.197*
BMI	0.120*	0.141*	0.247*	–0.043	–0.062	0.091*	0.153*	0.001
Frequency of going hungry^b^	–0.338*	–0.389*	–0.204*	–0.128*	–0.070	–0.312*	–0.152*	–0.111*
Number of people per room in house^c^	–0.187*	–0.385*	–0.019	–0.054	–0.131*	–0.136*	–0.414*	–0.117*

* Denotes a statistically significant correlation (p < 0.05)

^a^Highest level of schooling completed, categorised as 0 = none, 1 = primary, 2 = middle, 3 = high, 4 = college/technical, 5 = university. None of the mothers had been educated in Srinagar

^b^Self reported frequency of someone in the household going hungry for lack of money, categorised as 0 = never, 1 = occasionally, 2 = certain times of year, 3 = most months, 4 = most weeks, 5 = most days

^c^Number of people per room = number of people living in the house/number of rooms in the house [excluding kitchen and bathroom(s)]

**Fig. 4:**
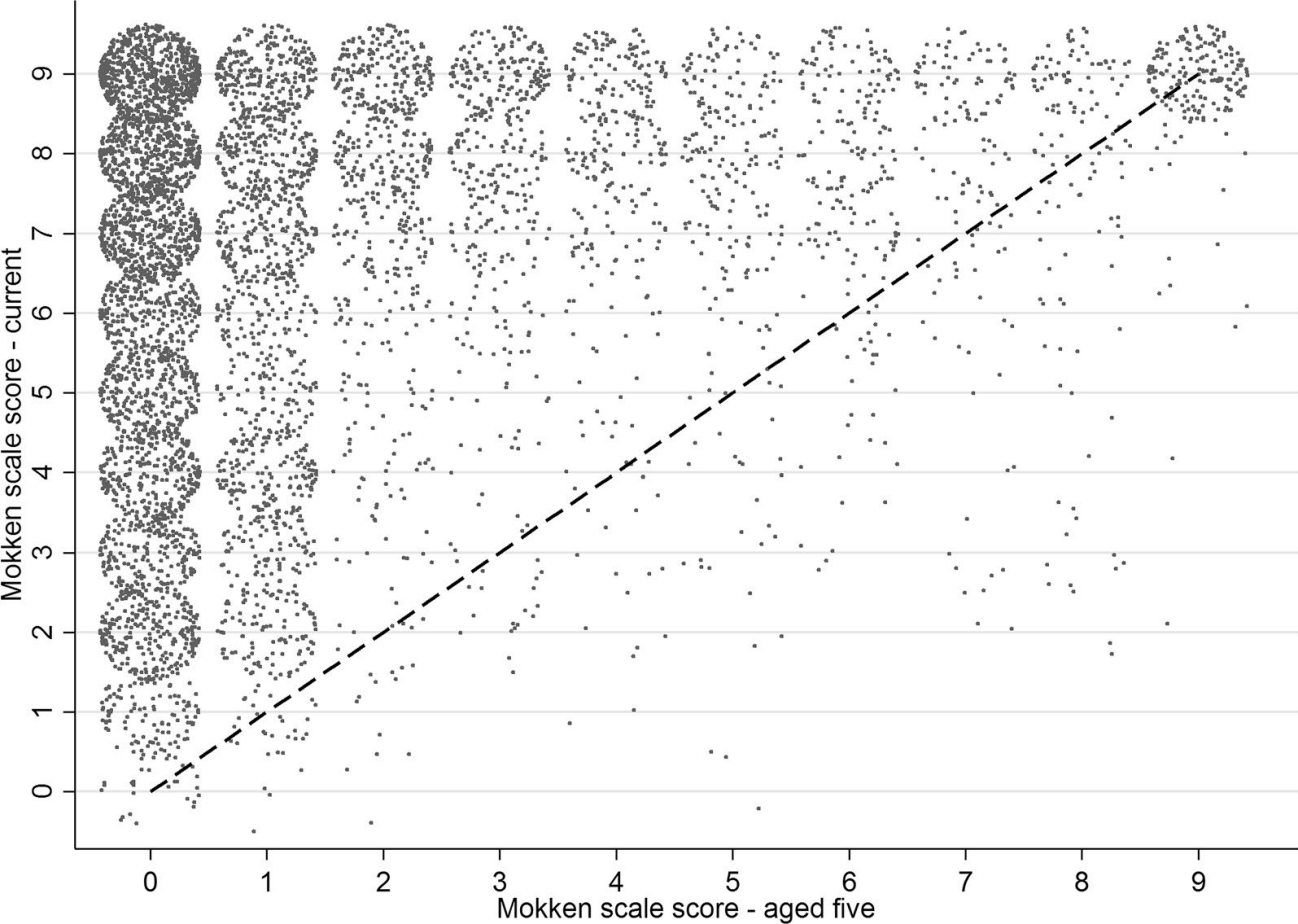
Mokken scale scores for current asset ownership vs. scores for aged 5 years. Some random noise has been added to the data to prevent many points overlying each other. 1:1 *line* is also shown. Note—cell phone was excluded from the current assets to make the scores more directly comparable with the scores for age 5

**Fig. 5:**
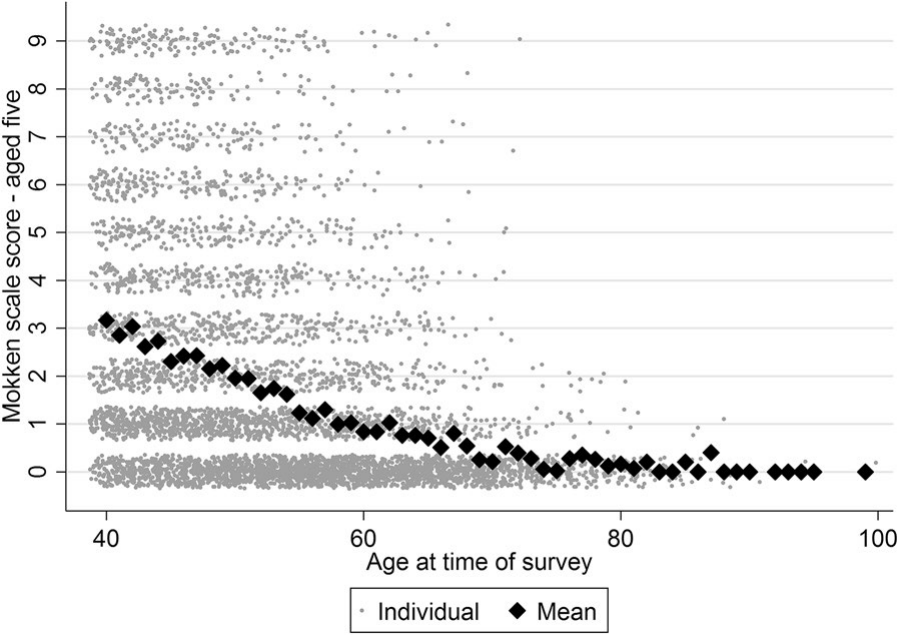
Association between respondents’ current age and their Mokken scale score for age 5 years. The *figure* relates the number of Mokken scale assets the respondent reported owning in their household when they were 5 years old to their age at the time of the survey. The mean scores for all respondents in each 1 year age group are also shown

Using the data on childhood ownership of the selected assets (excluding cell phone) the H coefficient was 0.70 indicating strong scalability also amongst these data. There was a general increase in the number of household assets owned in adulthood compared with childhood with only a small number of respondents having fewer assets in adulthood (Fig. 4). The number of assets in childhood decreased with increasing age of the respondent (Fig. 5), as would be expected.

**Fig. 6:**
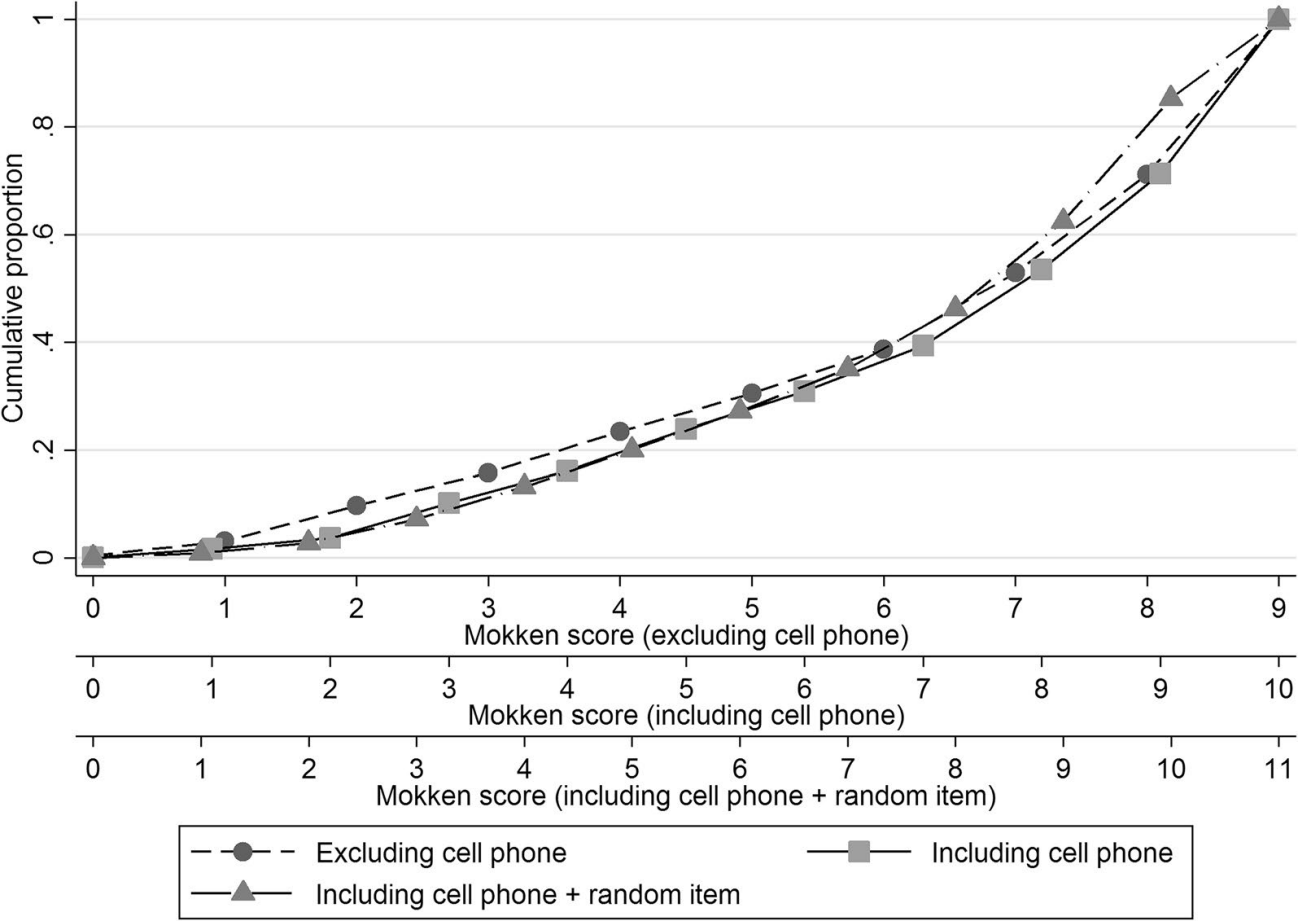
Cumulative distributions of scores for the current asset ownership data compared to the distributions when one item (cell phone) was omitted or and additional, random item was included

Sensitivity analyses showed that removing an item from the scale or adding an item uncorrelated to socio-economic position had little effect on the cumulative distribution of scores (Fig. 6). Individuals’ scores could only be decreased or increased by one point by these actions so despite inevitable differences in individual scores such changes would be unlikely to have a major effect on any apparent relationships between the scores and health outcomes. The Mokken scale scores were also robust to deleting and re-imputing some of the values. Even with 25 % of the data imputed, 67 % of individual scores were unchanged and 95 % were within ±1 of the original score (Table 4).

**Table 4:** Effects of imputing missing data

Error	5 % imputed	10 % imputed	25 % imputed
	N (%)	N (%)	N (%)
−4	0 (0.0)	0 (0.0)	1 (0.0)
−3	0 (0.0)	0 (0.0)	17 (0.2)
−2	6 (0.1)	27 (0.4)	126 (1.8)
−1	225 (3.2)	437 (6.3)	893 (12.8)
0	6423 (91.9)	5920 (84.7)	4691 (67.1)
1	316 (4.5)	560 (8.0)	1042 (14.9)
2	16 (0.2)	36 (0.5)	178 (2.5)
3	0 (0.0)	6 (0.1)	32 (0.5)
4	0 (0.0)	0 (0.0)	6 (0.1)
Total	6986 (100.0)	6986 (100.0)	6986 (100.0)
Number of respondents with ≥1 imputed response	2869 (41.1)	4621 (66.1)	6583 (94.2)
Number of respondents with error within ±1	6964 (99.7)	6917 (99.0)	6626 (94.8)

Errors in Mokken scale scores after removing a percentage of the responses for each asset at random and then re-imputing the data

Error was defined as the difference between the score using imputed data and the score using the original, observed data. The number and percentage of respondents with different magnitudes and directions of error are shown

Scores on the Mokken scale comprised of 10 assets were compared with scores on the first principal component obtained from PCA using either all 15 of the original assets, or just the 10 assets included in the Mokken scale. In both cases the PCA scores were very highly correlated with the Mokken scale scores (15 items, r_s_ = 0.977; 10 items, r_s_ = 0.996). As for the Mokken scale scores, mean PCA scores for each country showed strong rank correlation with GNI per capita (15 items, r_s_ = 0.929; 10 items, r_s_ = 0.905). The association between the gross national wealth and the mean participant asset index measured by either the Mokken Scale or by PCA was essentially the same.

## Discussion

Using an automated selection procedure it was found that the majority (10) of the 15 assets included in the questionnaire could be incorporated into a single scale such that the mean number of items owned was closely related to wealth as shown by the GNI per capita of the country. There was a high degree of consistency in the rank ordering of percent ownership of these items across countries, and the same set of assets was found to be at least weakly scalable in all of the study countries except the richest, Saudi Arabia, where most people owned most of the assets. Ownership of the set of assets also demonstrated an appreciable improvement in living standards for most people since the time they were aged five, as one might expect.

The set of assets used in this study were similar to those used in many other studies [1]. Although not all items were clearly related to socio-economic position in all of the countries, sensitivity analysis suggested that use of the scale would not be greatly affected by inclusion of items which were uncorrelated with socio-economic position in some countries or by dropping items to maintain comparability. Missing values could be imputed with reasonable accuracy suggesting the technique may be useful where missing data would require the exclusion of many individuals in other forms of multivariate analysis.

PCA has become a widely used method for estimating people’s socio-economic position rankings from an asset based inventory [1, 19–22]. However there have been criticisms about its applicability to binary data, leading some authors to propose using polychoric correlations in the calculations [23]. In this study we found this made negligible difference to the scores (Pearson’s correlation coefficient between scores calculated with and without using polychoric correlations was 0.999). Multiple correspondence analysis (MCA) has also been proposed [24] although the results are highly correlated with those from PCA [25]. More practical difficulties with using PCA are that the relationship between scores and asset ownership is usually unclear (for example different numbers of assets can sometimes lead to the same score) and comparison of scores between countries is complex [26, 27].

Zinn et al. [15] applied the Mokken scaling method to asset data in Indonesia in 1992 and found that a scale could be made up from selected assets. They argued that the technique was attractive because it was easy to see the relationship between wealth scores and the list of assets. Much later the method has also been tested in Vietnam [9]. The authors found it simple to apply and very similar in the way it ranks individuals to PCA, as we also observed.

In this study we found statistically significant differences in item functioning (DIF) implying that for people with the same score (which we considered to be a proxy for socio-economic position) the probability of owning any particular asset differed to some extent between countries. Whist this would preclude reliable comparisons of socio-economic position based on any particular asset, the score as a whole is a function of ownership of a set of assets which were acquired with a similar order of ability or preference in all of the countries.

We did not have any direct measure of socio-economic position at the individual or household level with which to compare the range of scores within countries. However we found positive correlations in most countries between the individuals’ scores and other variables collected for each respondent that might be expected to increase with increasing socio-economic positon (education, height and BMI). Conversely negative correlations were found between Mokken scale scores and the number of people per room in the house and the frequency of going hungry for lack of money as might be expected for a scale related to socio-economic position.

Despite the simplicity of Mokken scales, their ease of interpretation and apparent near equivalence to PCA, their uptake may have been hindered by a lack of familiarity or availability of suitable software. However routines are now readily available for Stata, R, SAS and other specialist software. The Mokken scaling software includes an automated item selection procedure and tests for goodness of fit so that a suitable set of assets can be chosen from amongst a longer list of candidate questions. For maximum efficiency, therefore, a list of potential questions might be included in a pilot survey and the list shortened accordingly before the main survey. Specialist software is only required for the development and testing of scales. Scoring an individual on an established scale is simply a matter of counting the number of the items that they own.

Mokken scaling provides a potentially useful way of assessing wealth on an ordinal scale and hence of testing whether health outcomes are related to socio-economic position. It could be used to distinguish rich from poor across a wide range of settings and therefore to help target healthcare where it is most needed. Reidpath and Ahmadi [9] highlighted the need for further studies to assess the method’s applicability across a range of countries. The BOLD study provided one such opportunity and we believe our results extend the evidence in support of the widespread usefulness of this method.

Since previous work had shown that GNI per capita had the greatest effect on lung function amongst the poorest countries [4], the assets included in our questionnaires were focussed on discriminating between levels of poverty mainly within developing countries. In the richer countries in our study most people owned most of the assets but this was not considered to be a significant limitation because we did not expect the level of wealth within rich countries to be a major influence on our outcomes of interest. For other types of study, however, it may also be useful to include questions about assets such as internet use and leisure activities to discriminate between people within the richer countries.

## Conclusions

Use of a Mokken scale appears to be very similar to PCA in its ability to discriminate between people of different socio-economic position but may have advantages in interpretability, adaptability and robustness to variations in the scale or missing data. Now that appropriate software is readily available its use should be considered as a possible alternative to other methods where it is required to rank individuals’ socio-economic position on the basis of an asset inventory.
